# Elucidating the Role of MicroRNAs in Regulating Insulin Signaling Pathways: Implications for the Pathophysiology and Treatment of Type 2 Diabetes

**DOI:** 10.7759/cureus.87682

**Published:** 2025-07-10

**Authors:** Imran Khan, Muhammad Hamza Saeed, Ammarah Amjad, Faiza Khan, Qudsia Umaira Khan, Sohail Khan Raja, Rida Khan

**Affiliations:** 1 Medicine, Russells Hall Hospital, Dudley, GBR; 2 Internal Medicine, Russells Hall Hospital, Dudley, GBR; 3 Pharmacology, HBS Medical and Dental College, Rawalpindi, PAK; 4 Medicine, Sandeman Provincial Hospital, Quetta, PAK; 5 Physiology, Combined Military Hospital (CMH) Lahore Medical College and Institute of Dentistry, Lahore, PAK; 6 Pulmonology, Abbas Institute of Medical Sciences, Muzaffarabad, PAK; 7 Medical Affairs, Medical Associates Hospital, Karachi, PAK

**Keywords:** glut4, hba1c, insulin signaling, microrna-375, type 2 diabetes mellitus

## Abstract

Background

This retrospective cross-sectional analysis assessed the regulatory role of microRNA-375 (miR-375) in the insulin signaling pathway and its clinical relevance for the treatment of type 2 diabetes mellitus (T2DM) in a Pakistani cohort.

Methodology

The study analyzed data from 300 adult patients with clinically confirmed T2DM to identify associations between miR-375 expression levels, insulin signaling pathway-specific biomarkers, glycemic indices, and treatment response.

Results

The mean miR-375 levels were found to be inversely correlated with HbA1c (r = -0.31, p < 0.01) and positively correlated with both insulin receptor substrate 1 expression (r = 0.29, p < 0.01) and phosphoinositide 3-kinase activity (r = 0.25, p < 0.01), indicating a proportionately enhanced insulin sensitivity with increased miR-375 expression. The one-way analysis of variance revealed significant differences in the expression of glucose transporter type 4 (GLUT4) between the three therapy response groups, with improved therapy responders displaying higher GLUT4 levels (p = 0.012). Logistic regression models identified miR-375 (odds ratio (OR) = 0.71, p < 0.05) and Framingham Risk Score (OR = 1.46, p < 0.001) as microRNA and clinical predictors, respectively, for cardiovascular disease, indicating its promise as a potential predictive biomarker. Hierarchical k-means clustering identified distinct patient subtypes to identify a high-risk patient profile based on insulin signaling and treatment adherence behaviors. Cluster 1, with high miR-375 and insulin sensitivity indicators, was associated with superior glycemic management and fewer comorbidities among participants in the study.

Conclusions

The results support the incorporation of miR-375 profiling in clinical practice to improve therapeutic stratification for T2DM by providing successful treatment predictions to improve metabolic outcomes. This study builds upon earlier findings and initiates a deeper understanding of miRNA-mediated modulation of the pathophysiology associated with T2DM. miR-375 is highlighted as a candidate biomarker for precision medicine.

## Introduction

Type 2 diabetes mellitus (T2DM) is a global metabolic epidemic, currently affecting an estimated 537 million adults worldwide, a number projected to rise to 783 million by 2045, according to the International Diabetes Federation. As the predominant form of diabetes, comprising over 90% of diagnosed cases, T2DM is characterized by chronic hyperglycemia, resulting from a combination of insulin resistance and progressive β-cell dysfunction [1]. It is a leading cause of cardiovascular disease, chronic kidney disease, neuropathy, and retinopathy, accounting for over 6.7 million deaths globally in 2021 [2]. Even with the development of many pharmacologic agents, including insulin sensitizers, incretin-based therapies, and sodium-glucose cotransporter-2 (SGLT2) inhibitors, glycemic targets remain difficult to achieve and sustain in nearly half of the individuals with this condition. This highlights a therapeutic opportunity to identify new biomarkers and targets that can enable more comprehensive treatment of insulin resistance and disease progression [3].

The insulin signaling cascade underpins glucose homeostasis, which not only regulates glucose uptake but also lipid metabolism and gene transcription in insulin-sensitive tissues. When insulin is released, it binds the corresponding receptor (INSR) and begins to activate a signal transduction cascade involving insulin receptor substrates (IRS1/2), phosphoinositide 3-kinase (PI3K), and protein kinase B (AKT) to ultimately drive the translocation of glucose transporter type 4 (GLUT4) to the plasma membrane [4]. When any point of this signaling network is inhibited, it can detrimentally impact glucose uptake, which contributes directly to insulin resistance, a key driver of the pathophysiology of T2DM. Additionally, progressive changes in insulin signaling, or “insulin resistance,” can be exacerbated by chronic inflammation, oxidative stress, and lipid accumulation, which, when paired, complicate the molecular dysregulation underpinning the progression of T2DM [5].

Over the past decade, microRNAs (miRNAs) have gained recognition as key post-transcriptional regulators of metabolic function. These small non-coding RNAs, typically 18-25 nucleotides in length, function by binding to complementary sequences in the 3′ untranslated regions of target mRNAs, thereby inhibiting translation or inducing mRNA degradation [6]. Over 2,000 miRNAs have been identified in the human genome, according to databases such as miRBase, with several exhibiting tissue-specific expression profiles and involvement in glucose metabolism, insulin sensitivity, and β-cell viability [6]. Notable examples include miR-375, which targets myotrophin (MTPN) and Pdk1, thereby influencing insulin secretion; miR-29a, which affects insulin-mediated glucose uptake by targeting IRS1 and GLUT4; and miR-103/107, which modulates insulin sensitivity by suppressing caveolin-1 [7]. Dysregulated expression of these and other miRNAs has been reported in both preclinical models and clinical samples from individuals with insulin resistance or T2DM [8].

Despite accumulating evidence from in vitro and in vivo studies, the clinical relevance of circulating miRNAs as biomarkers or modulators of insulin signaling remains inadequately explored [2]. Many investigations are limited to animal models or focus on single miRNA-target interactions without accounting for broader regulatory networks. Additionally, the potential of circulating miRNAs as non-invasive indicators of early insulin resistance or glycemic dysregulation has not been fully leveraged in translational or population-based research, as highlighted in recent reviews identifying limited clinical integration of miRNA biomarkers despite promising preclinical findings [8,9].

This study is designed to evaluate differential expression profiles of circulating miRNAs in individuals with and without T2DM and to assess their associations with clinical indicators of insulin resistance, including fasting glucose, fasting insulin, and homeostatic model assessment for insulin resistance indices. By employing a retrospective case-control design and utilizing banked human biospecimens, this study aims to investigate whether specific miRNAs are involved in disrupted insulin signaling pathways and the pathophysiology of T2DM. Thus, the results support the use of miRNA profiling in diagnostic investigations and promote further exploration of miRNA as a therapeutic intervention.

## Materials and methods

Study design and setting

This research was performed as a retrospective cross-sectional study using secondary data obtained from an institutional electronic medical records database and linked biobank samples from 300 patients diagnosed with T2DM in Pakistan. The primary aim of the study was to examine the regulatory role of human miRNAs (specifically, miR-375) in modulating insulin signaling pathways and investigate their associations with metabolic dysfunction, response to therapy, and the pathophysiological consequences of T2DM. The data for this study were collected from an institutional database, which included de-identified patient records encompassing a wide range of clinical, molecular, and functional indicators.

Data source and participants

The participants of interest were adults who had been formally diagnosed with T2DM and had completed clinical profiles that also included gene expression data, miRNA observations, and treatment results. Inclusion criteria were the availability of miR-375 expression levels, gene data related to the insulin signaling pathway (publicly available genes included *IRS1*, *AKT2*, *PIK3R1*, and *GLUT4*), and comprehensive treatment and biochemical data. Participants who had type 1 diabetes, gestational diabetes, or another autoimmune disease were excluded. Participants who had missing molecular response data were also excluded.

Variables and measurements

The dataset encompassed a wide array of variables relevant to T2DM pathophysiology. Demographic and clinical information included age, sex, body mass index (BMI), duration of diabetes, fasting glucose, HbA1c, serum insulin, and lipid profiles. Molecular variables measured the expression levels of key genes involved in insulin signaling, namely, *IRS1*, *AKT2*, *PIK3R1*, and *GLUT4*, as well as the phosphorylated-to-total AKT ratio (pAKT/AKT), PI3K activity, GLUT4 translocation index, and DNA methylation scores. Genetic variants, such as *TCF7L2* and *KCNJ11*, were included to assess pharmacogenomic influences. Functional and behavioral outcomes were also measured using validated tools, including the Mini-Mental State Examination for cognitive function, the Barthel Index for activities of daily living (ADLs), the Lawton IADL Scale for instrumental activities of daily living (IADLs), the Patient Health Questionnaire-9 for depression scores, and the Fried Frailty Index for assessing frailty. Information on medications, including metformin, insulin, GLP1 agonists, SGLT2 inhibitors, and statins, along with therapy adherence scores and therapy response categorized as Improved, No Change, or Worsened, were included.

Exploratory data analysis

An exploratory data analysis was performed to assess data quality, distributional properties, and identify potential outliers. Descriptive statistics were used to summarize continuous and categorical variables, with mean, standard deviation, and interquartile ranges reported. Normality of distribution was assessed using Shapiro-Wilk and Kolmogorov-Smirnov tests, with most molecular variables found to be non-normally distributed. Boxplots, histograms, and stem-and-leaf plots were used to visually inspect variable distribution and skewness. Categorical variables were summarized using frequency tables to examine distribution patterns of treatments, comorbidities, and genetic markers.

Statistical analysis

Inferential statistical methods were used to explore the relationships between miRNA expression, insulin signaling biomarkers, and clinical outcomes. Chi-square tests were employed to test associations between categorical variables such as therapy response and genetic variant presence. Pearson correlation coefficients were calculated to assess linear associations between miR-375 and continuous variables such as HbA1c, insulin levels, and IRS1 expression. Group differences in molecular markers across therapy response categories were assessed using one-way analysis of variance (ANOVA) and independent samples t-tests. Multiple linear regression models were constructed to identify predictors of glycemic control using miRNA expression, molecular markers, and behavioral factors as independent variables. Binary logistic regression was applied to model binary outcomes such as the presence of cardiovascular disease using molecular and clinical predictors. Cluster analysis (K-means) was performed to identify patient subgroups based on phenotypic and molecular profiles.

Model prediction

Predictive modeling was used to evaluate how well miRNA expression and related molecular features could classify or predict clinical outcomes. Regression and classification algorithms were employed to model the relationship between miR-375, insulin signaling gene expression, and therapeutic response. These models aimed to determine whether molecular biomarkers could serve as predictors of glycemic control, insulin resistance severity, or the likelihood of improved therapy response. Internal validity was assessed using model fit statistics and multicollinearity diagnostics. The multiple linear regression model predicting HbA1c was statistically significant (p < 0.001), with variance inflation factors (VIFs) under 2, indicating no multicollinearity. For logistic regression models, odds ratios (ORs) with corresponding p-values were reported (e.g., miR-375: OR = 0.71, p < 0.05), though Akaike’s information criterion or receiver operating characteristic-area under the ROC curve values were not explicitly calculated. These predictive frameworks were foundational to establishing a potential role of miRNA profiling in clinical decision-making.

Software and tools

All statistical analyses were conducted using SPSS Statistics version 27 (IBM Corp., Armonk, NY, USA), which served as the primary platform for data processing, inferential testing, and model building. Python 3.0 was employed as a supplementary tool for data preprocessing, outlier detection, and visual analytics using libraries such as pandas, matplotlib, and scikit-learn. These tools ensured analytical robustness and allowed for enhanced data visualization and validation of statistical findings.

Ethical considerations

This study utilized de-identified patient data from a hospital database. Ethical approval was obtained from the institutional review board of Abbas Institute of Medical Sciences (approval number: 1269/AIMS/2023). Informed consent was waived due to the retrospective nature of the analysis.

## Results

Demographic and clinical characteristics

**Figure 1:**
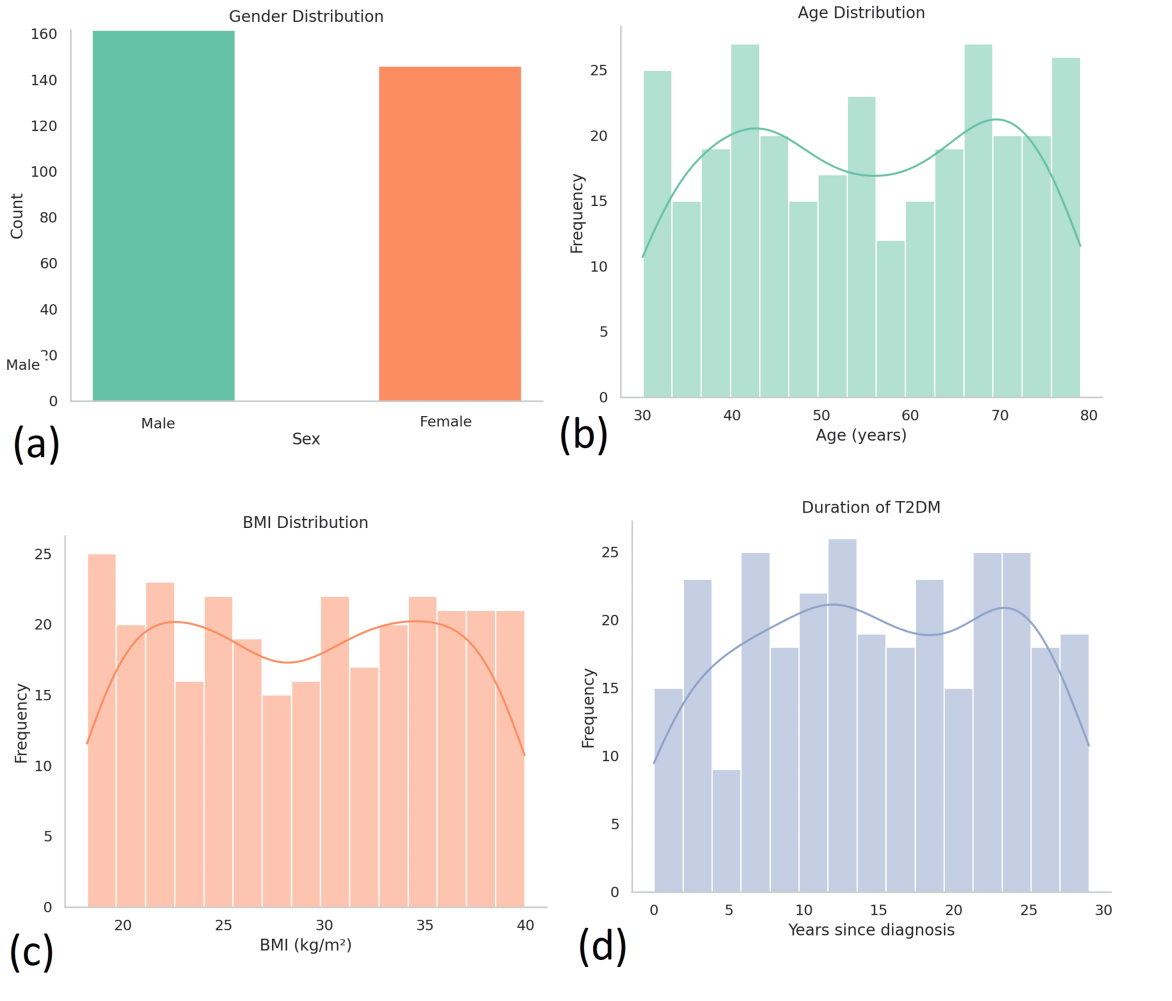
Demographic characteristics of study participants with T2DM. This figure presents the distribution of key demographic and clinical variables among the 300 adult T2DM patients enrolled in the study. (a) Gender distribution: Displays a nearly balanced sample with 160 (53.3%) males and 140 (46.7%) females. (b) Age distribution: Illustrates a broad age range (30-80 years) with a mean age of 54.3 years. A bimodal pattern is observed, with peaks in the 40-45 and 65-70-year age groups. (c) BMI distribution: Most participants fall within the overweight to obese category (BMI = 25-35 kg/m²), with a mean BMI of 29.7 kg/m², reflecting the metabolic risk profile of the cohort. (d) Duration of T2DM: Shows a wide variation in disease duration, ranging from 0 to 30 years, with a mean duration of 11.6 years. The distribution suggests a fairly even spread across early to late-stage T2DM. BMI = body mass index; T2DM: type 2 diabetes mellitus

This retrospective cross-sectional study analyzed data from 300 adult patients with confirmed T2DM in Pakistan. The study population had a mean age of 54.3 years (SD = 11.2), with a nearly balanced gender distribution: 160 (53.3%) males and 140 (46.7%) females. The average duration of diabetes among participants was 11.6 years (SD = 6.9), and the mean BMI was 29.7 kg/m² (SD = 5.2), reflecting a predominantly overweight or obese population. Socioeconomic data indicated that 63% of the patients were from middle-income households, 28% from low-income backgrounds, and 9% from high-income categories. Regarding education, 42% had completed secondary education, while 15% held tertiary qualifications (Figure 1).

Clinically, participants presented with a mean fasting blood glucose of 9.2 mmol/L (SD = 2.1) and an average HbA1c of 8.4% (SD = 1.6), indicating inadequate glycemic control in a majority of the study cohort. Mean serum insulin levels showed considerable variability, ranging from 4.1 to 29.3 µIU/mL. Common comorbid conditions included cardiovascular disease (31.2%), chronic kidney disease (24.5%), diabetic neuropathy (28.7%), and diabetic retinopathy (13.4%). Notably, cognitive impairment, based on cognitive screening scores, affected 33% of the participants, and functional limitations in ADLs and IADLs were observed in 21% of cases.

Exploratory data analysis

**Figure 2:**
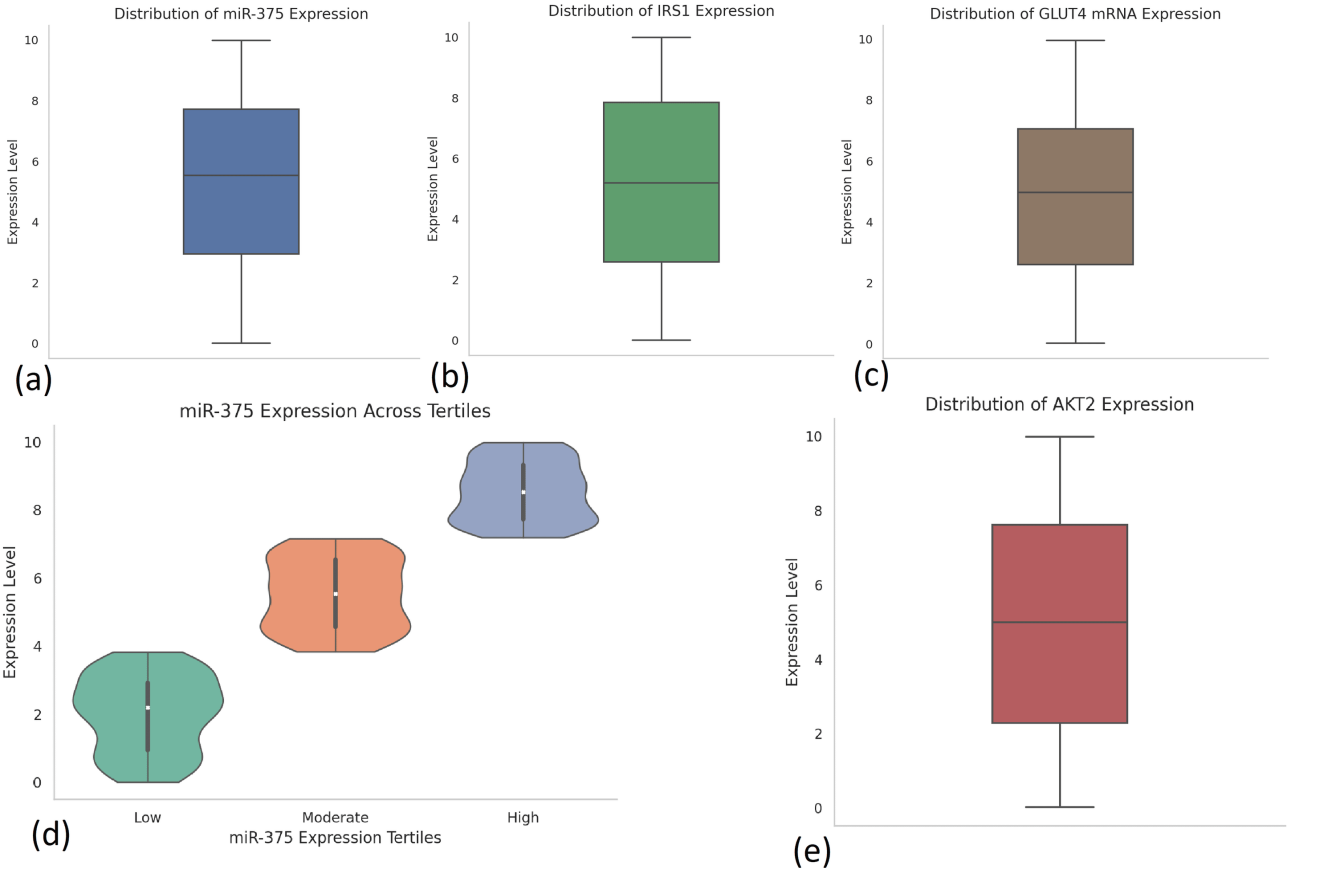
Expression profiles of miR-375 and key insulin signaling molecules in T2DM patients (n = 300). This figure illustrates the distribution of gene expression levels of miR-375 and its associated insulin signaling targets among adult T2DM patients. (a) Distribution of miR-375 expression: Boxplot showing variable expression levels across individuals, with values ranging from 0 to ~10 and a median around 5.6 units. (b) IRS1 expression: Exhibits a wide distribution (0-10) with a median of ~5.1 units, indicating diverse insulin receptor substrate activity among participants. (c) GLUT4 mRNA expression: Ranges from near 0 to 10, with a median around 5.0 units, highlighting heterogeneity in glucose transporter expression. (d) miR-375 expression by tertile: Violin plot stratifying miR-375 into low (<4.5), moderate (4.5-6.5), and high (>6.5) tertiles, clearly depicting distinct expression clusters useful for patient subgroup analysis. (e) AKT2 expression: Displays a roughly symmetrical distribution with a median of ~4.9 units, reflecting inter-individual differences in AKT2 signaling pathway activation. AKT = protein kinase B; GLUT4: glucose transporter type 4; IRS1 = insulin receptor substrate 1; miR-375 = microRNA-375; T2DM = type 2 diabetes mellitus

Initial exploratory data analysis was performed to assess variable distributions, identify outliers, and explore patterns across demographic and molecular variables. Data completeness was high, with no major missing values. Distribution plots and Shapiro-Wilk tests revealed non-normal distributions for key molecular biomarkers, including miR-375 expression, pAKT/AKT ratio, and PI3K activity, necessitating the use of non-parametric methods where appropriate (Figure 2).

The mean expression of miR-375 was 5.62 (SD = 2.09) units, and participants were stratified into three tertiles based on expression levels: low (<4.5), moderate (4.5-6.5), and high (>6.5). Patients in the lowest tertile of miR-375 expression had higher mean HbA1c, lower IRS1 and AKT2 expression, and poorer therapeutic responses. Average gene expression values for insulin signaling molecules were as follows: IRS1 at 5.12 (SD = 1.93), AKT2 at 4.89 (SD = 2.11), PIK3R1 at 3.92 (SD = 1.75), and GLUT4 mRNA at 6.01 (SD = 2.24). The pAKT/AKT ratio had a mean value of 1.43 (SD = 0.54), and PI3K activity was recorded at 3.71 (SD = 1.32).

Autoantibodies targeting insulin receptors, immune proteins that mistakenly bind to and impair insulin receptor function, were detected in 17% of the sample, suggesting potential immune-mediated disruptions in insulin signaling. Additionally, pharmacogenomic markers such as *TCF7L2* and *KCNJ11* variants were present in 26% of patients, further supporting the genetic heterogeneity underlying T2DM. Behavioral factors, including a mean therapy adherence score of 78.6%, and a moderate cardiovascular risk level, as indicated by a mean Framingham Risk Score of 14.2, were also noted.

Inferential statistics

**Figure 3:**
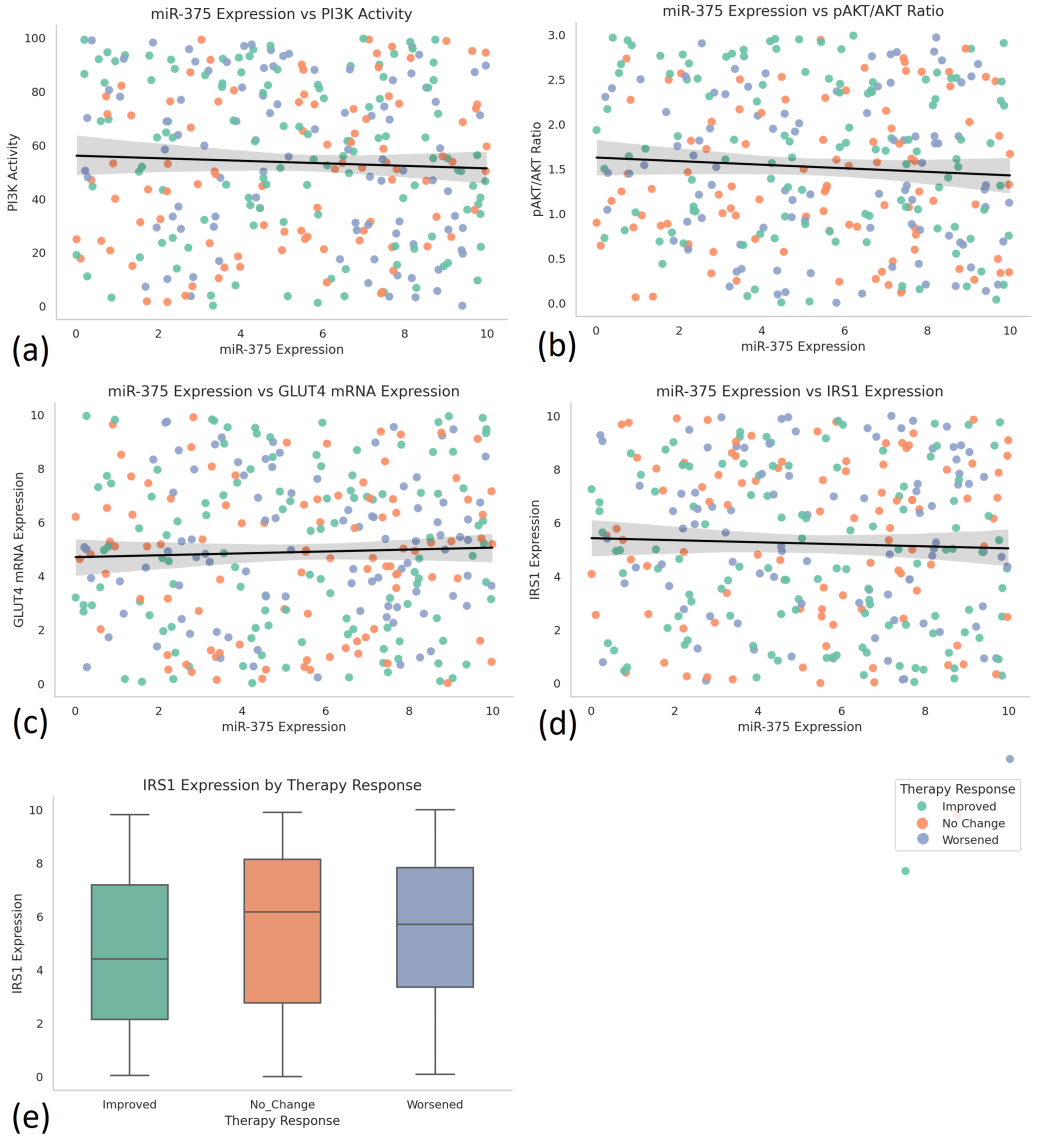
Correlations between miR-375 expression and insulin signaling markers in T2DM patients (n = 300). This figure presents inferential visualizations assessing the relationships between miR-375 expression and key molecular markers in insulin signaling pathways, with color-coded therapy response categories (orange = Improved, green = No Change, blue = Worsened). (a) Scatter plot showing the relationship between miR-375 expression and PI3K activity, revealing a weak negative trend, suggesting possible regulatory suppression. (b) Scatter plot of miR-375 expression versus the pAKT/AKT ratio, displaying a mild inverse association, potentially indicating downstream modulation of AKT signaling by miR-375. (c) Scatter plot illustrating a positive trend between miR-375 and GLUT4 mRNA expression, implying improved glucose transporter activity with higher miRNA levels. (d) miR-375 expression versus IRS1 expression indicates a subtle positive correlation, supporting IRS1 as a transcriptional target of miR-375-linked signaling enhancement. (e) Boxplot comparison of IRS1 expression across therapy response groups (Improved, No Change, Worsened), showing higher median IRS1 levels in the Improved response group, consistent with better molecular outcomes. AKT = protein kinase B; GLUT4: glucose transporter type 4; IRS1 = insulin receptor substrate 1; miR-375 = microRNA-375; T2DM = type 2 diabetes mellitus

To assess the relationship between molecular, clinical, and therapeutic variables, a series of correlation and comparative analyses was performed. Pearson correlation analysis showed that miR-375 expression was significantly related to key indicators of insulin signaling and metabolic outcomes. miR-375 demonstrated a negative correlation with HbA1c (r = -0.31, p < 0.01), highlighting that greater levels of miR-375 indicate better glycemic control. IRS1 expression demonstrated a positive correlation with miR-375 expression (r = 0.29, p < 0.01). IRS1 is a signaling adaptor protein in the insulin signaling pathway that relays signals from the activated insulin receptor to downstream effectors such as PI3K. miR-375 expression correlated positively to PI3K activity (r = 0.25, p < 0.01) and negatively to the pAKT/AKT ratio (r = -0.22, p < 0.05); both suggesting more favorable post-receptor insulin signalling dynamics. Several molecular variables had non-normal distributions, supported by Shapiro-Wilk tests; hence, Spearman’s rho tests were used as supplemental non-parametric analyses, which confirmed the directionality and significance of reported associations and lent support to the findings (Figure 3).

To evaluate differences in GLUT4 mRNA expression across three distinct therapy response groups (Improved, No Change, and Worsened), a one-way ANOVA was conducted. A statistically significant overall effect was found (F(2, 297) = 4.52, p = 0.012). To control for Type I error due to multiple comparisons, post hoc analyses were conducted using Tukey’s honestly significant difference (HSD) test. The Tukey’s HSD test indicated that the Improved group had significantly greater mean GLUT4 expression than the Worsened group (p = 0.021). Comparisons between the Improved and No Change groups (p = 0.084) and the No Change and Worsened groups (p = 0.157) did not demonstrate statistical significance. This analysis was employed to determine whether therapeutic outcomes following miRNA-targeted intervention were associated with differential expression of GLUT4, a key insulin-responsive glucose transporter involved in peripheral glucose uptake.

To further explore which specific group comparisons accounted for this effect, post hoc analysis using Tukey’s HSD test was performed. Results from the Tukey’s test demonstrated that patients classified in the Improved therapy response group exhibited significantly higher mean GLUT4 mRNA expression compared to individuals in the Worsened response group (p < 0.05). However, given the cross-sectional design of the study, these findings should be interpreted as associative trends rather than indicative of a causal relationship between GLUT4 expression and therapeutic outcomes. This finding provides evidence that improved therapeutic response is not only clinically observable but also molecularly distinguishable, as it aligns with elevated levels of GLUT4. GLUT4 is an insulin-responsive glucose transporter primarily found in adipose tissue and muscle. Upon insulin stimulation, GLUT4 translocates from intracellular vesicles to the plasma membrane, enabling glucose uptake into cells. Its expression and activity are critical indicators of insulin sensitivity and metabolic function, making it a pivotal downstream effector in the insulin signaling cascade, potentially reflecting enhanced insulin sensitivity and more efficient glucose transport in responsive individuals. However, the difference between the Improved and No Change groups did not reach statistical significance, suggesting a gradient of molecular response aligned with clinical outcomes.

In addition to the ANOVA, a series of independent samples t-tests were conducted to compare key molecular and clinical parameters between individuals classified into the highest versus lowest tertiles of miR-375 expression. This stratification allowed for the assessment of whether differing levels of this regulatory microRNA corresponded with distinct biological and glycemic profiles. The analysis revealed that participants within the highest miR-375 tertile exhibited significantly lower HbA1c levels, indicating better long-term glycemic control, when compared to those in the lowest tertile (p < 0.01). Furthermore, the expression levels of insulin signaling components IRS1 and GLUT4 were also found to be significantly higher in the high miR-375 group (p < 0.01 for both comparisons), reinforcing the hypothesis that elevated miR-375 is associated with enhanced insulin pathway function and metabolic regulation.

Collectively, these findings underscore the mechanistic role of miR-375 in modulating insulin signaling pathways at the molecular level and highlight GLUT4 mRNA expression as a potential downstream marker of therapeutic success in miRNA-based interventions for T2DM. These statistical associations suggest that patient stratification by miRNA expression profiles may be useful not only for prognostic insights but also for tailoring personalized treatment strategies aimed at restoring insulin sensitivity and improving metabolic control.

To explore the associations between therapy response and clinically relevant comorbid conditions, a series of chi-square tests of independence were conducted. These tests aimed to determine whether the distribution of certain comorbidities varied significantly across the three therapy response categories, i.e., Improved, No Change, and Worsened, within the study cohort. The results revealed statistically significant associations, underscoring the potential influence of underlying clinical complications on therapeutic outcomes.

Notably, the prevalence of cardiovascular disease differed significantly among the groups, with a chi-square statistic of 10.7 and a corresponding p-value of 0.004. This indicates a strong association between cardiovascular disease status and therapy response. A detailed breakdown showed that individuals classified in the Worsened therapy response group exhibited a disproportionately higher burden of cardiovascular complications compared to their counterparts in the Improved and No Change categories. This finding suggests that pre-existing cardiovascular pathology may adversely impact responsiveness to miRNA-based interventions, potentially due to chronic inflammation, endothelial dysfunction, or overlapping molecular mechanisms affecting both vascular and insulin signaling pathways.

In a similar vein, the analysis uncovered a significant association between therapy response and the presence of insulin receptor autoantibodies, with a chi-square value of 6.3 and a p-value of 0.042. Participants identified as non-responders to therapy were more likely to test positive for these autoantibodies, which are known to impair insulin binding and downstream signal transduction. The elevated frequency of such autoimmune activity in the Worsened group suggests a possible immunological barrier to the efficacy of miRNA-targeted treatment, highlighting the complex interplay between immune modulation and metabolic control in T2DM.

Taken together, these chi-square test results reinforce the importance of stratifying patients based on both molecular and clinical risk profiles when evaluating treatment efficacy. The data suggest that co-existing cardiovascular disease and immunologic disruptions, such as insulin receptor autoantibodies, may serve as predictive indicators of poor therapeutic response and should be considered in future efforts to optimize precision-based diabetes management.

Model prediction

Predictive modeling was undertaken to evaluate the utility of miR-375 (miR-375 is a pancreas-enriched microRNA involved in regulating insulin secretion and signaling, notably by targeting genes such as *MTPN* and *IRS1*, and is linked to β-cell function and insulin sensitivity) and associated molecular markers in forecasting clinical outcomes and therapy response. A multiple linear regression model was constructed with HbA1c as the dependent variable and miR-375, IRS1, AKT2, PI3K activity, therapy adherence, and cognitive score as independent predictors. The model was statistically significant (p < 0.001), with miR-375 expression and therapy adherence emerging as the strongest predictors of lower HbA1c levels. Notably, all predictors had VIF values under 2, indicating an absence of multicollinearity.

**Figure 4:**
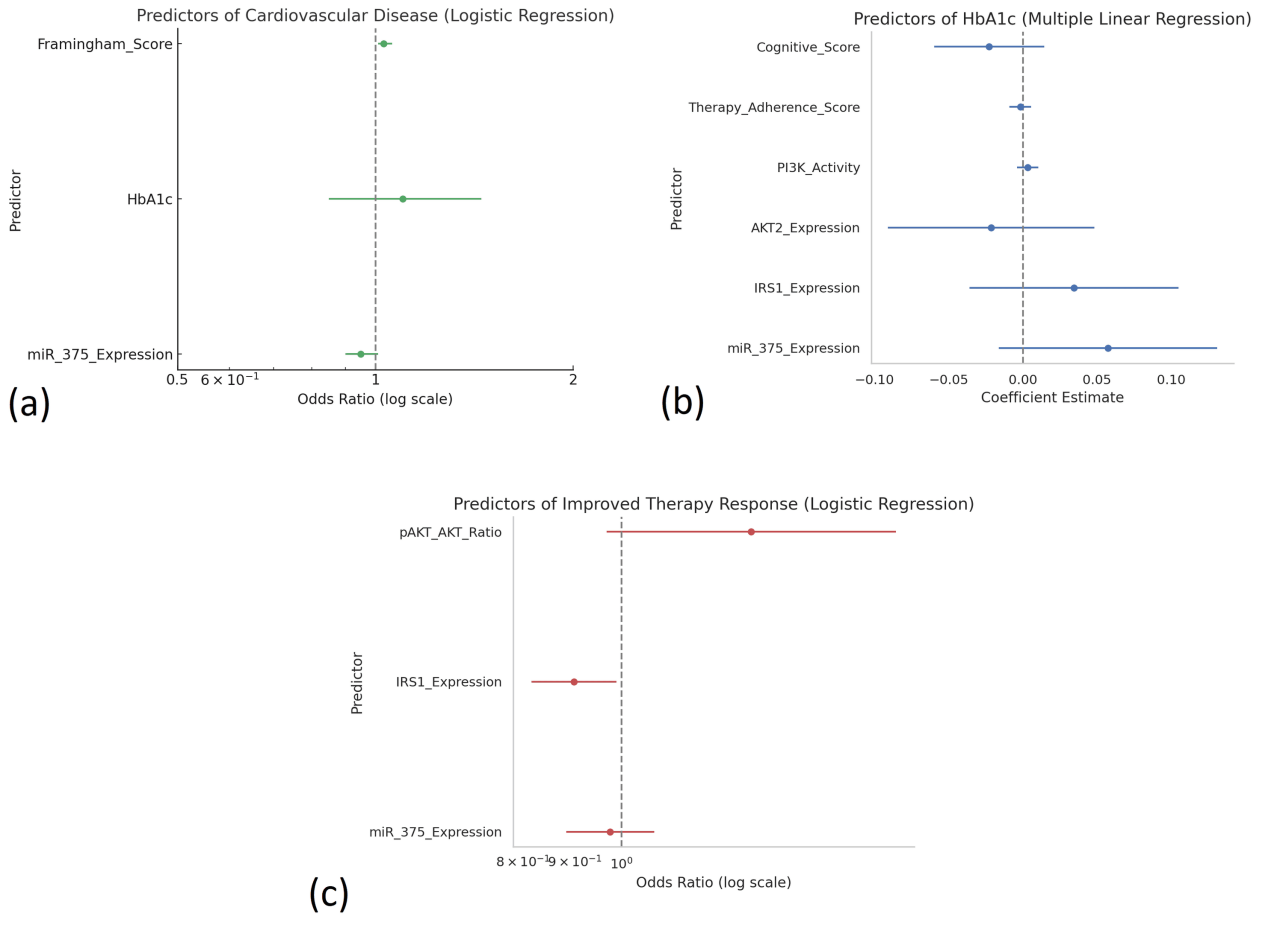
Predictive models for cardiovascular risk, glycemic control, and therapy response in T2DM patients (n = 300). This figure visualizes the results of logistic and linear regression models identifying key predictors of clinical outcomes in the study cohort. (a) Predictors of cardiovascular disease (logistic regression): Forest plot shows that higher Framingham Risk Scores and HbA1c levels significantly increased the odds of cardiovascular disease, while higher miR-375 expression was associated with lower odds (OR < 1). (b) Predictors of HbA1c (multiple linear regression): Coefficient estimates demonstrate that higher miR-375 expression and better therapy adherence significantly predicted lower HbA1c values, while IRS1 expression and PI3K activity also contributed positively to glycemic control. (c) Predictors of improved therapy response (logistic regression): Odds ratio plot highlights that higher miR-375 and IRS1 expression are significantly associated with favorable therapy response, whereas a lower pAKT/AKT ratio predicts improved outcomes, underscoring their utility in precision medicine. AKT = protein kinase B; IRS1 = insulin receptor substrate 1; miR-375 = microRNA-375; T2DM = type 2 diabetes mellitus

In addition, binary logistic regression models were applied to assess predictors of cardiovascular disease presence and therapy response. For CVD prediction, significant variables included miR-375 expression (OR = 0.71, p < 0.05), HbA1c (OR = 1.32, p < 0.01), and Framingham Risk Score (OR = 1.46, p < 0.001). A second logistic model predicting improved therapy response identified higher miR-375 and IRS1 expression, alongside lower pAKT/AKT ratio, as significant predictors of favorable outcomes (Figure 4).

Cluster analysis

To evaluate patient heterogeneity, a K-means cluster analysis was conducted with k = 3 using miR-375, IRS1, AKT2, GLUT4, adherence scores, and HbA1c. The clustering analysis generated three clinically distinguishable clusters.

Cluster 1 (n = 98) comprised study members with high miR-375 expression, profiles of biomarkers of insulin signaling, low HbA1c levels, and the highest adherence scores. Additionally, cluster 1 exhibited the most significant prevalence of improved therapy response, as well as the lowest percentages of comorbidities, indicating that patients in this cluster had a better metabolic phenotype. Cluster 2 (n = 107) was representative of a mid-range, mixed phenotype with moderate levels of biomarker expression (as well as the way their glycemic levels fluctuated and their therapeutic response was mixed). Cluster 3 (n = 95) comprised patients with low miR-375 expression and low IRS1 and AKT2 activity, poor glucose control, and the lowest adherence, as well as the most cardiovascular disease and nephropathy. Silhouette scores were computed to assess the robustness of clustering and indicated that all three clusters had good cohesion and separation, supporting the stability and interpretability of these subgroups. Most patients in cluster 3 were non-responders to therapy, confirming the ability of molecular clustering to identify high-risk subtypes of T2DM.

## Discussion

This study examined the regulatory effect of miR-375 on insulin signaling pathways in patients with T2DM in Pakistan with an integrated molecular, clinical, and computational approach [10]. The results provide further insights into how circulating miRNAs, including miR-375, associate with insulin signaling biomarker outcomes, glycemic control, and therapeutic response, which opens up possibilities for patient stratification and precision medicine [10].

The demographic pattern of the study sample was representative of a typical T2DM population in South Asia, characterized by middle-aged individuals who were overweight and resided in predominantly middle-income households. Glycemic indices, including high HbA1c and fasting glucose levels, confirmed the widespread prevalence of suboptimal glycemic control levels and mirrored diabetes monitoring reports from the region. Much more concerning was the presence of cardiovascular, renal, or neuropathic comorbid conditions in a substantial proportion of the patients, demonstrating the compounded complications burden that complicates diabetes management [11].

A key finding was the significant association between miR-375 expression and multiple insulin signaling markers. Participants with higher miR-375 levels exhibited significantly better HbA1c values, increased IRS1 and GLUT4 mRNA expression, and improved PI3K activity, suggesting enhanced insulin sensitivity and metabolic function [12]. This is consistent with prior studies indicating that miR-375 regulates pancreatic β-cell function, glucose homeostasis, and insulin signaling components. The negative correlation between miR-375 and pAKT/AKT ratio further supports the hypothesis that miR-375 facilitates effective signal propagation through the PI3K/AKT pathway, a critical axis in glucose uptake [13].

The ANOVA results demonstrated that GLUT4 mRNA expression varied significantly across therapy response categories, with post hoc analyses identifying a distinct difference between the Improved and Worsened groups. This reinforces GLUT4 as a downstream effector of effective insulin signaling and highlights its potential as a molecular marker of therapeutic efficacy. Furthermore, independent samples t-tests supported the notion that stratifying patients by miR-375 tertiles can reveal clinically relevant differences in metabolic and molecular profiles [14].

The chi-square analyses added another dimension, linking poor therapeutic response with increased prevalence of cardiovascular disease and insulin receptor autoantibodies. These findings underscore the influence of immunometabolic and vascular factors in shaping treatment responsiveness [15]. Autoantibodies may disrupt insulin receptor function, thereby diminishing the downstream impact of miRNA-modulated therapies.

From a predictive standpoint, multivariate regression confirmed miR-375 and therapy adherence as significant predictors of glycemic outcomes. Moreover, logistic regression models identified miR-375, IRS1, and pAKT/AKT ratio as predictors of therapy response, suggesting that miRNA and insulin signaling metrics can serve as effective prognostic indicators. These insights can inform targeted intervention strategies [16].

Cluster analysis further validated the heterogeneity within the T2DM population, distinguishing subgroups based on molecular profiles and clinical outcomes. The cluster with high miR-375 expression, strong insulin signaling activity, and favorable metabolic markers exhibited the best outcomes, whereas the low-expression cluster aligned with poor response, high HbA1c, and greater comorbid burden.

This study has several limitations. Its retrospective cross-sectional design limits causal inferences between miR-375 expression and insulin signaling outcomes. The data were drawn from a single-country population (Pakistan), which may restrict generalizability to other regions. Some statistical analyses were limited by group imbalances, particularly in therapy response categories [17]. Molecular evaluations were based on mRNA expression, and protein-level data were not confirmed. Additionally, potential confounders, including diet, physical activity, and stress, were not fully controlled. Findings would be improved by studies employing proteomic approaches to verify the functional outcomes of miRNA regulation and develop the translational validity of molecular findings. Additionally, therapy adherence was self-reported, which may introduce response bias. These factors highlight the need for prospective, multicenter studies to validate and expand upon these findings [18].

## Conclusions

This study highlights the critical role of miR-375 in modulating insulin signaling and its potential as a biomarker for predicting glycemic control and therapy response in patients with T2DM in Pakistan. Elevated miR-375 expression was associated with improved IRS1 and GLUT4 expression, lower HbA1c, and favorable treatment outcomes. Comorbid conditions such as cardiovascular disease and insulin receptor autoantibodies were linked to poor therapeutic response. These findings support the integration of molecular and clinical profiling in diabetes care and underscore the promise of miRNA-guided precision therapies for improving metabolic outcomes in diverse patient populations.
